# Supplementary material for: Stress-driven photo-reconfiguration of surface microstructures via vectorial field-guided lithography
Source: Light Sci Appl. 2026 Apr 10;15:194. doi: 10.1038/s41377-025-02174-5 (PMC13068957; doi:10.1038/s41377-025-02174-5)
Supplement: Supplementary file 1 — Supplementary Information [file 41377_2025_2174_MOESM1_ESM.pdf]

Supplementary information for

Stress-driven photo-reconfiguration of surface microstructures via vectorial field-guided lithography

I Komang Januariyasa,[1] Francesco Reda,[1] Nikolai Liubimtsev,[2] Pawan Patel,[2,3] Cody Pedersen,[4] Fabio Borbone,[5] Marcella Salvatore,[1] Marina Saphiannikova,[2,3,*] David McGee,[4,*] and Stefano Luigi Oscurato[1,*]

[1]Physics Department “E. Pancini”, University of Naples Federico II, Complesso Universitario di Monte Sant’Angelo, via Cinthia, 80126, Naples, Italy.

[2]Division Theory of Polymers, Leibniz Institute of Polymer Research Dresden, 01069 Dresden, Germany.

[3]Faculty of Mechanical Science and Engineering, Dresden University of Technology, 01062 Dresden, Germany.

[4] Department of Physics, The College of New Jersey, Ewing, New Jersey 08628, USA.

[5]Department of Chemical Sciences, University of Naples “Federico II”, Complesso Universitario di Monte Sant’Angelo, Via Cintia, 80126 Naples, Italy.

[*]Stefano Luigi Oscurato, e-mail: stefanolugi.oscurato@unina.it

[*]Marina Saphiannikova, e-mail: grenzer@ipfdd.de

[*]David J. McGee, e-mail: mcgeed@tcnj.edu

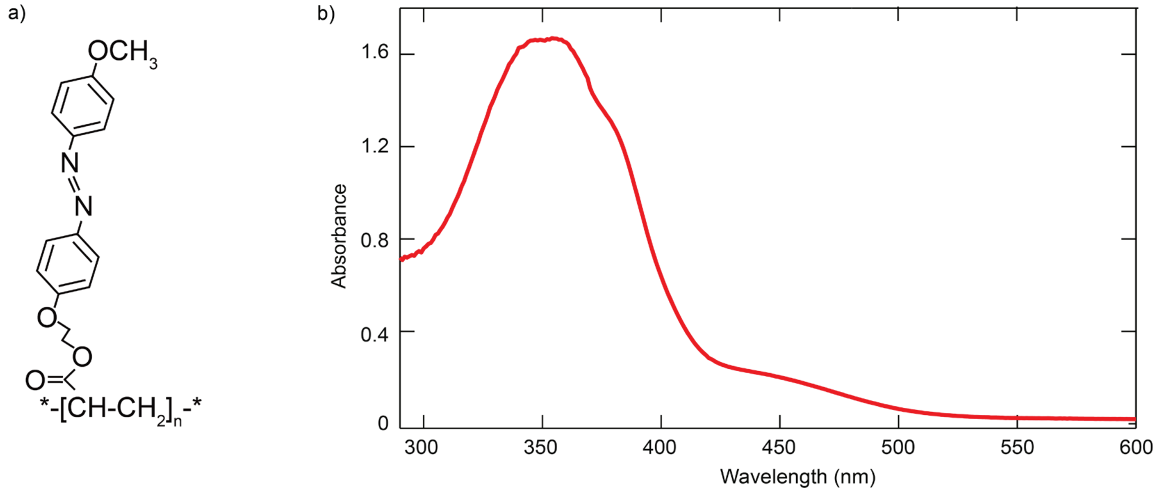

Figure S1. a) The chemical structure and b) the UV-Visible absorption spectrum of the azopolymer used in this study, recorded with Jasco V560 spectrophotometer.

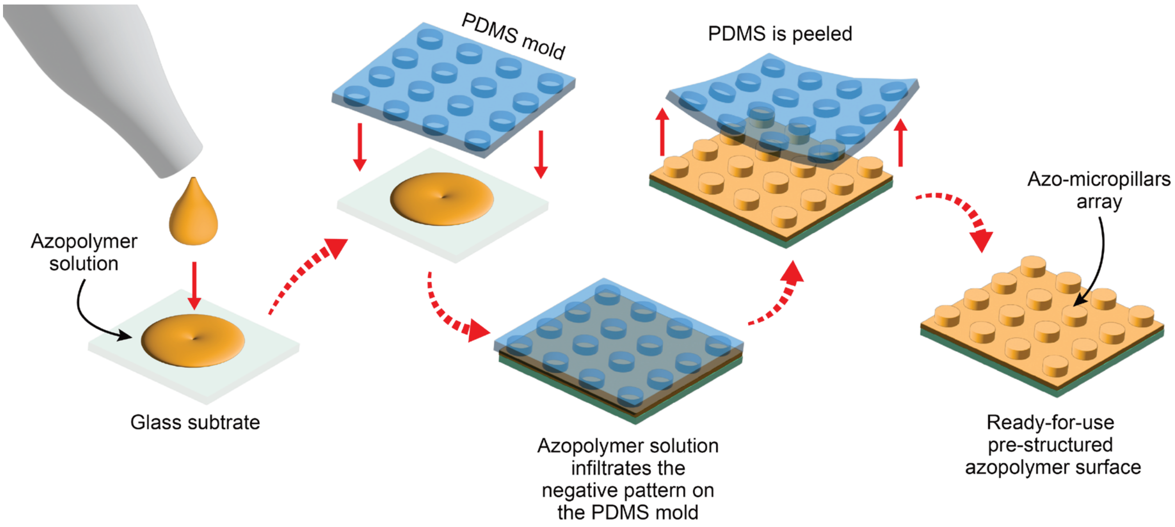

Figure S2. The procedure of the soft-lithography used to fabricate azo-micropillars array on the surface. A few drops of 10 wt% azopolymer solution in 1,1,2,2-tetrachloroethane was deposited onto a standard glass microscope coverslip. Then, the prepared PDMS stamp was gently placed onto the azopolymer solution, allowing the azopolymer solution to infiltrate the negative pattern on the stamp. The sample is then left at ambient temperature for approximately 5 hours, during which the solvent in the azopolymer solution evaporates completely. The final step is to carefully peel the PDMS stamp from the azopolymer surface, leaving the pre-structured azopolymer surface on the glass substrate to be used in the experiment. The pre-structured azo-micropillars can be selected to have a height (H_o), diameter (D_o), and periodicity (P_o) which are varied depending on the conducted experiment.

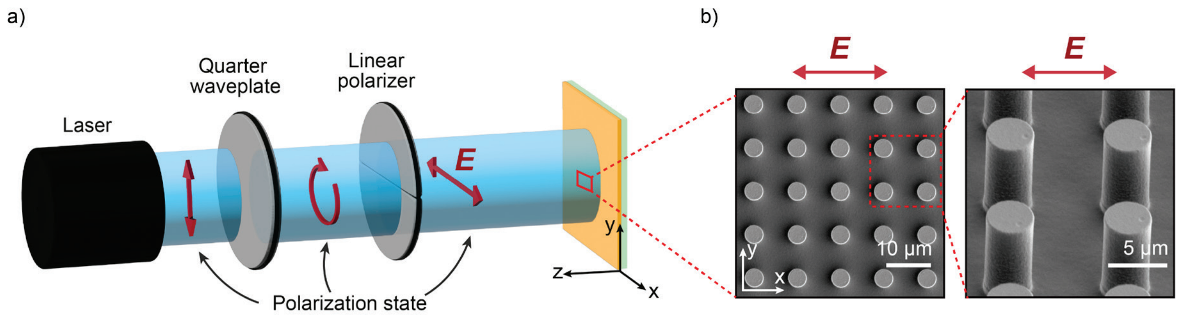

Figure S3. The illumination study of homogeneous beam with linear polarization. a) The optical configuration of the illumination exposure. The experimental study of homogeneous linearly polarized light irradiation was performed using a 488 nm laser (Coherent OBIS 488 LS) with the intensity on the surface plane set to about 160 mW cm^{-2}. b) The morphology of the pristine state of the azo-micropillar observed from top-down and inclined view. The measured pristine azo-micropillars have a height of $H_o \approx 8.4$ µm, diameter of $D_o \approx 4$ µm, and periodicity of $P_o \approx 10$ µm.

Supplementary note 1: Viscoplastic PhotoAlignment (VPA) modeling

1. Light-induced stress and orientation of backbone segments

The light induced stress $\boldsymbol{\tau}$ in the VPA modeling is defined by the rate of change of the 2nd order orientation tensor $\langle \mathbf{uu} \rangle$ of rigid segments of the polymer backbone:[1]

$$\boldsymbol{\tau} = 3nkT\lambda_{\mathrm{R}} \frac{\partial}{\partial t}\langle \mathbf{uu} \rangle \cdot \tag{S1}$$

Here $\mathbf{u}$ is the unit orientation vector of the segment, n is the number density of segments and λ_{R} is their rotational time in the absence of light. The orientation state of rigid backbone segments is described by the 2nd and 4th order orientation tensors $\langle \mathbf{uu} \rangle$ and $\langle \mathbf{uuuu} \rangle$. The time evolution of 2nd order tensor can be calculated as follows:

$$\frac{\partial}{\partial t}\langle \mathbf{uu} \rangle = -\frac{5V_r}{6\lambda_{\mathrm{R}}}\left\{\hat{\mathbf{E}}\hat{\mathbf{E}} \cdot \langle \mathbf{uu} \rangle + \langle \mathbf{uu} \rangle \cdot \hat{\mathbf{E}}\hat{\mathbf{E}} - 2\hat{\mathbf{E}}\hat{\mathbf{E}} : \langle \mathbf{uuuu} \rangle\right\} - \frac{\langle \mathbf{uu} \rangle}{\lambda_{\mathrm{R}}} + \frac{\boldsymbol{\delta}}{3\lambda_{\mathrm{R}}}, \tag{S2}$$

where $\hat{\mathbf{E}}$ is the unit vector of light polarization and $\boldsymbol{\delta}$ is the unit tensor. The parameter

$$V_r = 2qmV_0 / 5kT \tag{S3}$$

is the reduced strength of orientation potential U_{eff} acting on the backbone segments irradiated with linearly polarized light:

$$U_{\mathrm{eff}} = qmV_0\left(\hat{\mathbf{E}} \cdot \mathbf{u}\right)^2. \tag{S4}$$

Here V_0 is the strength of orientation potential, which effectively describes the gradual orientation of azobenzene chromophores after several photoisomerization cycles. A derivation of effective orientation potential from kinetic equations for the photoisomerization dynamics between trans and cis-isomers of azobenzenes can be found in Ref. 2. Furthermore, it is assumed that each of the backbone segments contains m azobenzenes, whose orientation distribution with respect to the long axes of the segments is described by the shape factor q. For side chain azopolymers q is close to -0.5,[3] which means that reorientation of azos perpendicular to the light polarization is transferred to an alignment of the polymer backbones along the same polarization direction. Note that the reduced strength V_r takes negative values because $q < 0$.

2. Homogeneous irradiation with linearly polarized light

The side-chain azopolymers align and stretch along the light polarization direction. Let's choose this direction as the axis x of local coordinate system (LCS). Under homogeneous irradiation, i.e. at the constant light intensity, the time evolution of the xx-component of the 2nd order orientation tensor is given by:

$$\frac{\partial}{\partial t}\langle u_x^2\rangle = \frac{1}{\lambda_{\mathrm{R}}}\left[V_r\left(\langle u_x^2\rangle - 1\right)\langle u_x^2\rangle - \langle u_x^2\rangle + \frac{1}{3}\right] \tag{S5}$$

This expression was derived in Supporting Information to Ref. 4. Due to the axial symmetry with respect to the light polarization, two other diagonal components are equal to each other:

$$\langle u_y^2\rangle = \langle u_z^2\rangle = \left(1 - \langle u_x^2\rangle\right)/2 \tag{S6}$$

and all off-diagonal components are equal to zero. Together with equation (S1), this means that the light induced stress $\boldsymbol{\tau}$ is also diagonal and symmetric around the axis x in LCS:

$$\tau_{yy} = \tau_{zz} = -\tau_{xx}/2\,. \tag{S7}$$

Hence, the tensile component of stress tensor $\tau_E = \tau_{xx}$ and the compressive components $\tau_\perp = -\tau_E/2$. The time-dependent magnitude of τ_E is calculated from equation (S1) using equations (S5) and (S6) with the initial value of $\langle u_x^2\rangle$= 1/3 which corresponds to isotropic orientation of backbone segments. The rotational time of polymer backbone segments is chosen to be long enough ($\lambda_{\mathrm{R}} = 1000$ s) to achieve non-vanishing stress values in the VPA modeling of azopolymer micropillars. The reduced potential strength V_r in equation (S5) is prescribed by the magnitude of light induced tensile stress at the beginning of irradiation:[4]

$$\tau_E(0) = -\frac{2}{3}nkTV_r\,. \tag{S8}$$

By implementing the light induced stress tensor into the custom subroutine Userthstrain of the finite element software ANSYS, we are able to reproduce the uniaxial deformation of a homogeneously irradiated azopolymer micropillar. For that we apply the visco-plastic Perzyna model, described by equation (3) in the main text. More details about the implementation of light induced stress can be found in the PhD thesis of B. Yadav.[5]

3. Light induced stress at the initial stages of irradiation

The light induced stress in the VPA model is described by rather complicated tensorial equations (S1) and (S2), which contain the dyadic tensor $\hat{\mathbf{E}}\hat{\mathbf{E}}$ and the orientation tensors $\langle \mathbf{uu} \rangle$ and $\langle \mathbf{uuuu} \rangle$. This makes difficult for a non-theorist to comprehend a relation between the light induced stress and the light polarization. Fortunately, it is possible to find an elegant solution to this system of tensor equations, assuming that the initially isotropic state of polymer backbones changes very slowly under light irradiation. Under this condition, the light induced stress remains close to its value at the beginning of irradiation.

First, we immediately see that the last two terms in equation (S2) cancel each other out, since the initially isotropic state is described by $\langle \mathbf{uu} \rangle = \boldsymbol{\delta}/3$. To simplify the expression in parentheses, it is necessary to apply a closure approximation to the 4th order orientation tensor $\langle \mathbf{uuuu} \rangle$. It is known that linear closure provides an exact solution for the random orientation of rods:[6]

$$\langle \mathbf{uuuu} \rangle = -\tfrac{1}{35}\boldsymbol{\Sigma}_4 + \tfrac{1}{7}\langle \mathbf{uu} \rangle \cdot \boldsymbol{\Sigma}_4 + \tfrac{1}{7}\boldsymbol{\Sigma}_4 \cdot \langle \mathbf{uu} \rangle \qquad \text{(S9)}$$

where $\boldsymbol{\Sigma}_4 = \boldsymbol{\delta\delta} + \mathrm{I} + \mathrm{I}^+$ represents the sum of 4th order isotropic tensors. Applying the linear closure to the expression in parentheses of equation (S2) results in

$$\hat{\mathbf{E}}\hat{\mathbf{E}} \cdot \langle \mathbf{uu} \rangle + \langle \mathbf{uu} \rangle \cdot \hat{\mathbf{E}}\hat{\mathbf{E}} - 2\hat{\mathbf{E}}\hat{\mathbf{E}} : \langle \mathbf{uuuu} \rangle = \frac{2}{5}\hat{\mathbf{E}}\hat{\mathbf{E}} - \frac{2}{15}Tr(\hat{\mathbf{E}}\hat{\mathbf{E}})\boldsymbol{\delta} \qquad \text{(S10)}$$

While the trace of $\hat{\mathbf{E}}\hat{\mathbf{E}}$ is equal to 1 for any polarization state,

$$\frac{\partial}{\partial t}\langle \mathbf{uu} \rangle = -\frac{2}{15\lambda_R}\frac{qmV_0}{kT}\left(\hat{\mathbf{E}}\hat{\mathbf{E}} - \boldsymbol{\delta}/3\right) = -\frac{V_r}{3\lambda_R}\left(\hat{\mathbf{E}}\hat{\mathbf{E}} - \boldsymbol{\delta}/3\right). \qquad \text{(S11)}$$

After substituting the time derivative of $\langle \mathbf{uu} \rangle$ into equation (S1),

we arrive to the equation (1) of the main text:

$$\boldsymbol{\tau} = \tau_0\left(\hat{\mathbf{E}}\hat{\mathbf{E}} - \boldsymbol{\delta}/3\right)$$

where $\tau_0 = -nkTV_r$ is the magnitude of light induced stress at the beginning of irradiation.

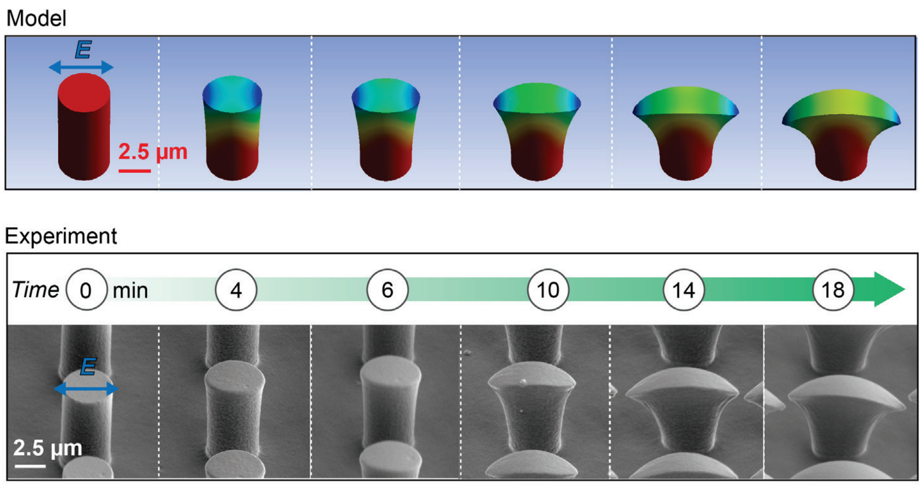

Figure S4. The basic study of the azo-micropillar deformation from a homogeneous linearly polarized beam. The morphological evolution of azo-micropillar from the model simulation (top row) and the experiment (bottom row) at different time points (0 – 18 min).

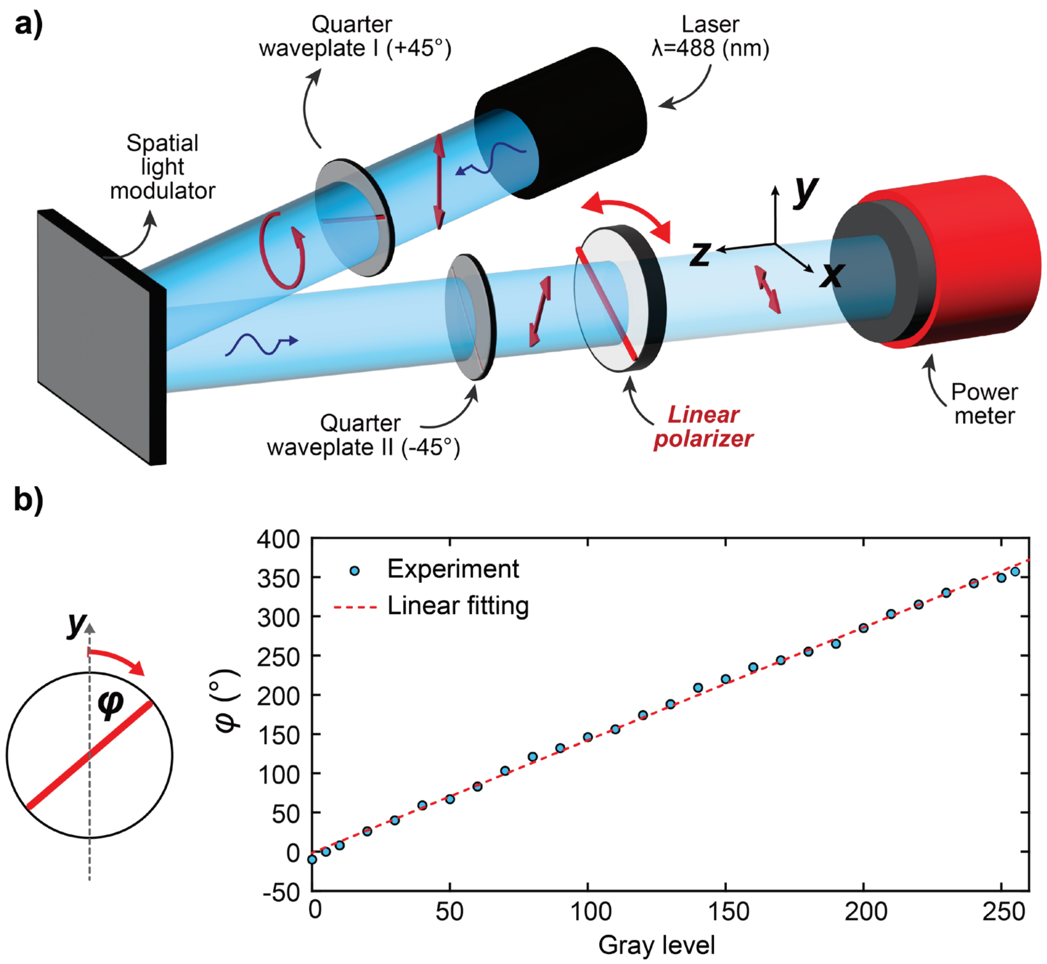

Figure S5. a) The optical setup for characterizing the digital polarization rotator. A characterization procedure was conducted to establish a conversion between the grayscale value in the image and the relative rotation of the polarization angle relative to a designated reference axis (the y-axis in this study). The procedure of this experiment is to input an image of uniform grayscale level (8-bit, 1920 pixels × 1152 pixels) into the spatial light modulator (SLM), which covered the entire active area, and then to measure the angle of the polarization axis (φ; relative to the optical y-axis) when the power meter measured the maximum power output. This angle represented the major axis of polarization relative to the y-axis. The process is repeated for the entire range of grayscale values (i.e., from 0 to 255). b) The curve of conversion grayscale to polarization rotation and its linear fitting.

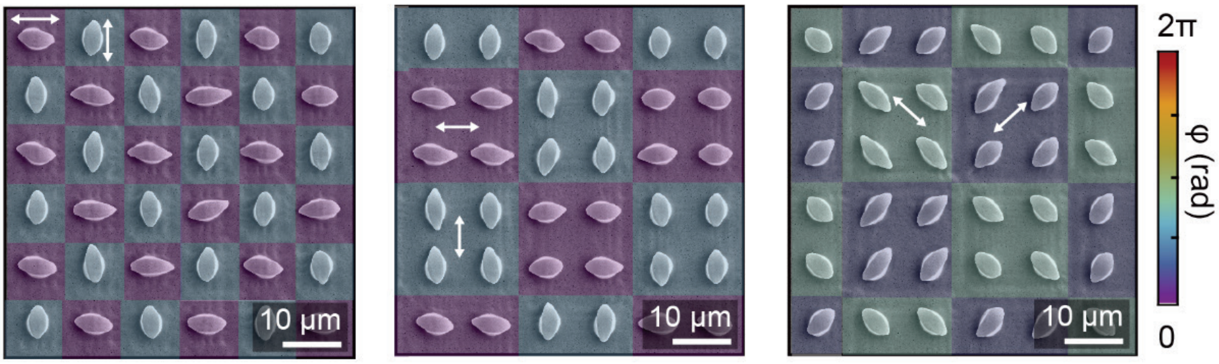

Figure S6. Surface reconfigurations with various checkerboard-styled polarization map designs, e.g., vertical-horizontal and diagonal with different numbers of micropillars for each polarization box.

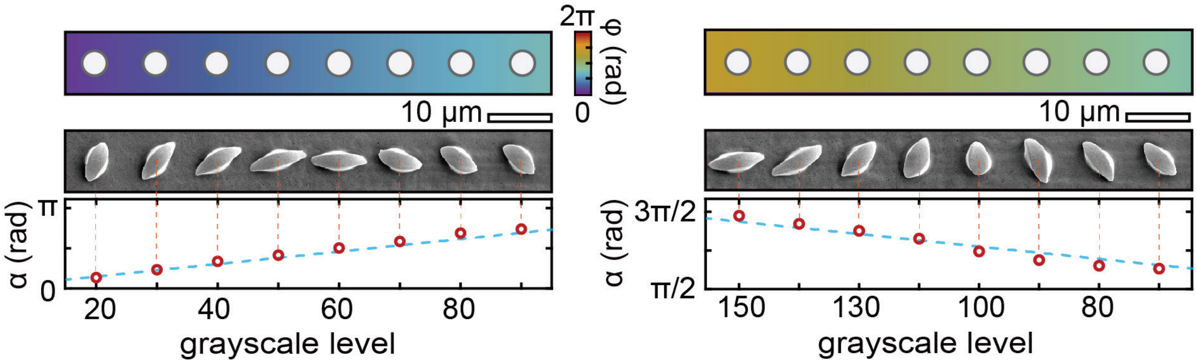

Figure S7. Surface reconfigurations with a gradual rotation of polarization azimuth across a row of micropillars. Each reconfiguration was produced with a random grayscale range (e.g., gradually increasing and decreasing trend in left and right data, respectively). α is the angle between the major axis of the micropillar elongation relative to *y*-axis in clockwise rotation. The correlation between the linearly varying polarization pattern and the resulting deformation orientation of the exposed micropillars was studied by measuring the deformation angles α as a function of the linearly increasing gray value assigned to the SLM. The analysis, shown in plot of gray level vs. α, shows that we achieved an excellent agreement with the expected picture of the gradually rotating stress pathways deforming the surface microstructures along the local polarization direction.

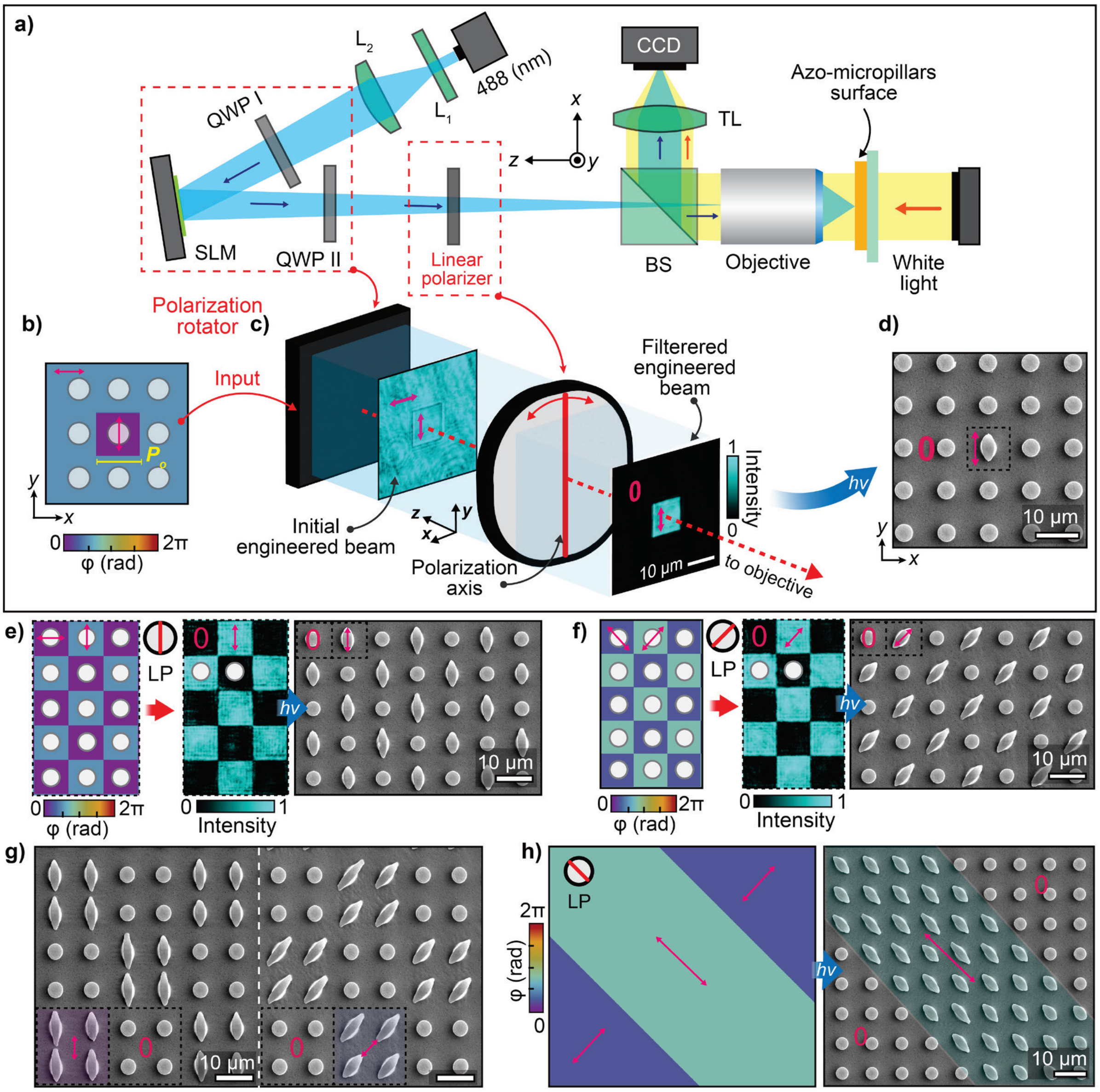

Figure S8. The introduction of the "polarization filter" strategy to impart a simple intensity-polarization control in the beam for complex surface configuration. a) The optical configuration of polarization rotator used in this study with an additional linear polarization after the polarization rotator part (i.e., after QWP II). b) The polarization map comprising a rectangular region exhibiting polarization rotation in the vertical direction ($\varphi = 0$), designed to occupy an area encasing a single micropillar ($P_o \times P_o$). The remaining portion of the polarization map is set to possess a polarization rotation in the horizontal direction ($\varphi = 90°$). c) The conceptual workflow of the polarization filter. When the polarization axis of linear polarizer is set to align with the polarization direction of the box region set in (b), it only allows the light of this part to pass and blocks the surrounding beam, producing a box pattern of intensity with vertical polarization direction. This filtered engineered beam provides a spatial selectivity, for example producing d) a deformation only to a single micropillar without affecting the others. The strategy can be expanded to more complex cases, such as a periodic configuration with its own directionality and number of reconfigured pillars (e–g) and an arbitrary pattern (h).

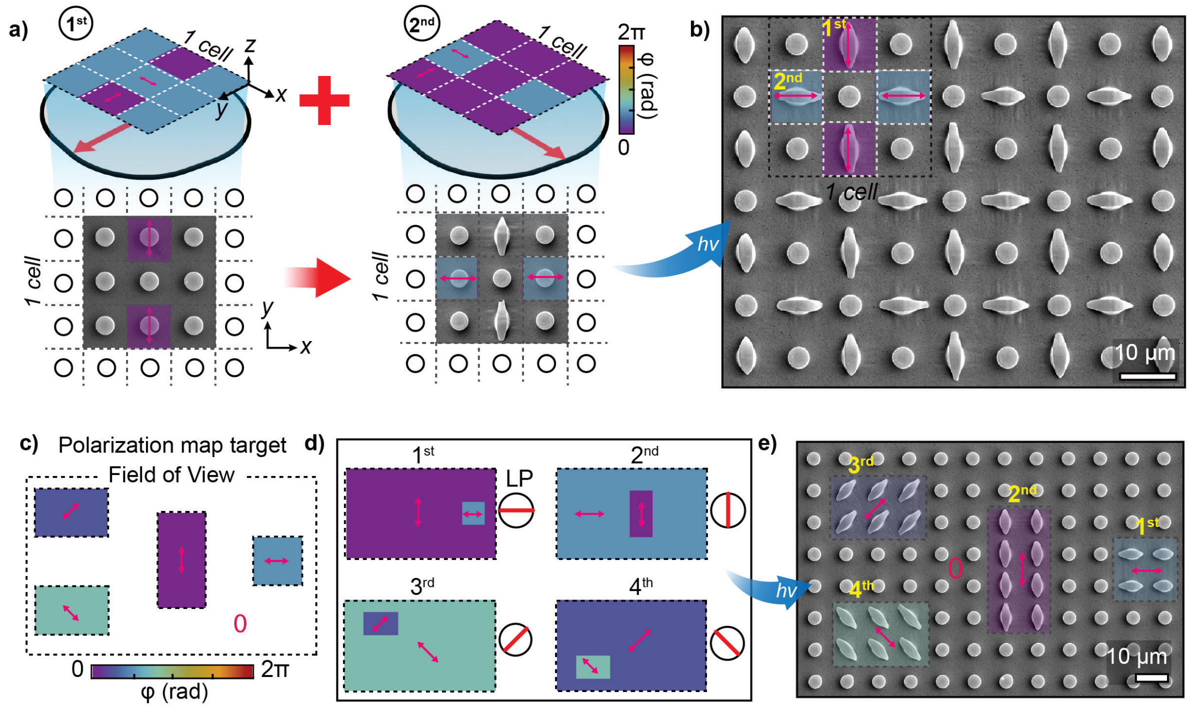

Figure S9. The implementation of the "multi-exposure" approach to achieve complex control of spatial selectivity and local directionality in the final surface configuration. a) The conceptual illustration of the 2-exposure approach to produce a periodic quad-petal formation of anisotropic microstructures. The 1st exposure is performed to generate a unidirectional element of the petals (i.e., the petals along the *y*-axis) while leaving an azo micropillar in the center of the petal formation unchanged. The 2nd exposure is then performed to add the other unidirectional element of the petals. b) The final morphology of the quad-petal formation of microstructures array. A demonstration of the multi-exposure approach to produce any configuration of anisotropic surface, such as a) 4-box areas with different sizes and local directionalities. To achieve this pattern, the key is to divide the map target into d) four different exposures. Each exposure is designed to convey one element (i.e., one box) of the target map by designing a polarization map that contains only the intended element of the target map and setting the rest of the field of view to orthogonal polarization. The polarization axis of the linear polarization is then adjusted to filter the engineered beam to have only the intended element of the target polarization. e) The final anisotropic surface configuration that matches the target polarization map.

Supplementary note 2: Visualization of light-induced stress pathways using ANSYS software

Primary deformation of azopolymer micropillars follows the major principal direction of the maximum tensile component of the photoinduced stress, which is dictated by the local polarization state of the incident light. In Figure S10, the direction of the x-axis in the local coordinate system for each finite element is shown. It is aligned with the major principal axis of the stress tensor, as the incident light field in this case is linearly polarized, producing a consistent spatial distribution of tensile stress pathways.

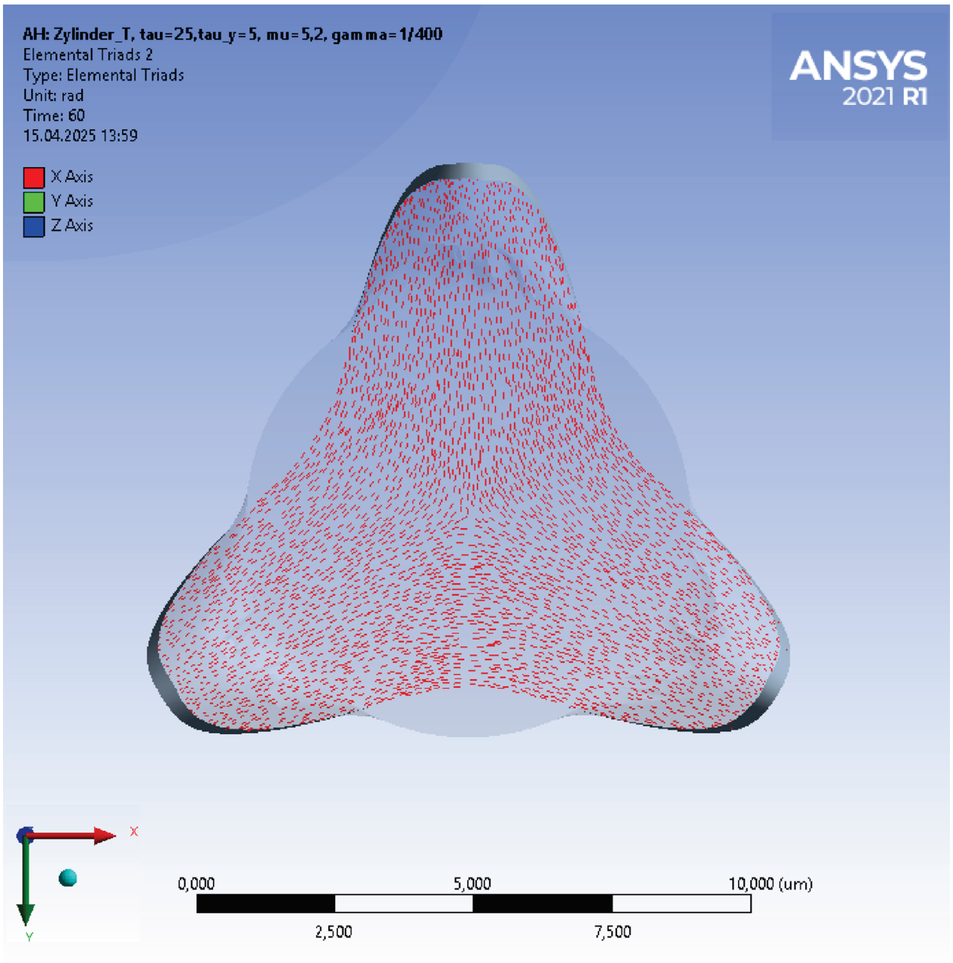

Figure S10. Orientation of the x-axis (red arrows) in the local coordinate system for each finite element, aligned with the major principal axis of the stress tensor. The distribution of axes is shown for the triaxial micropillar system.

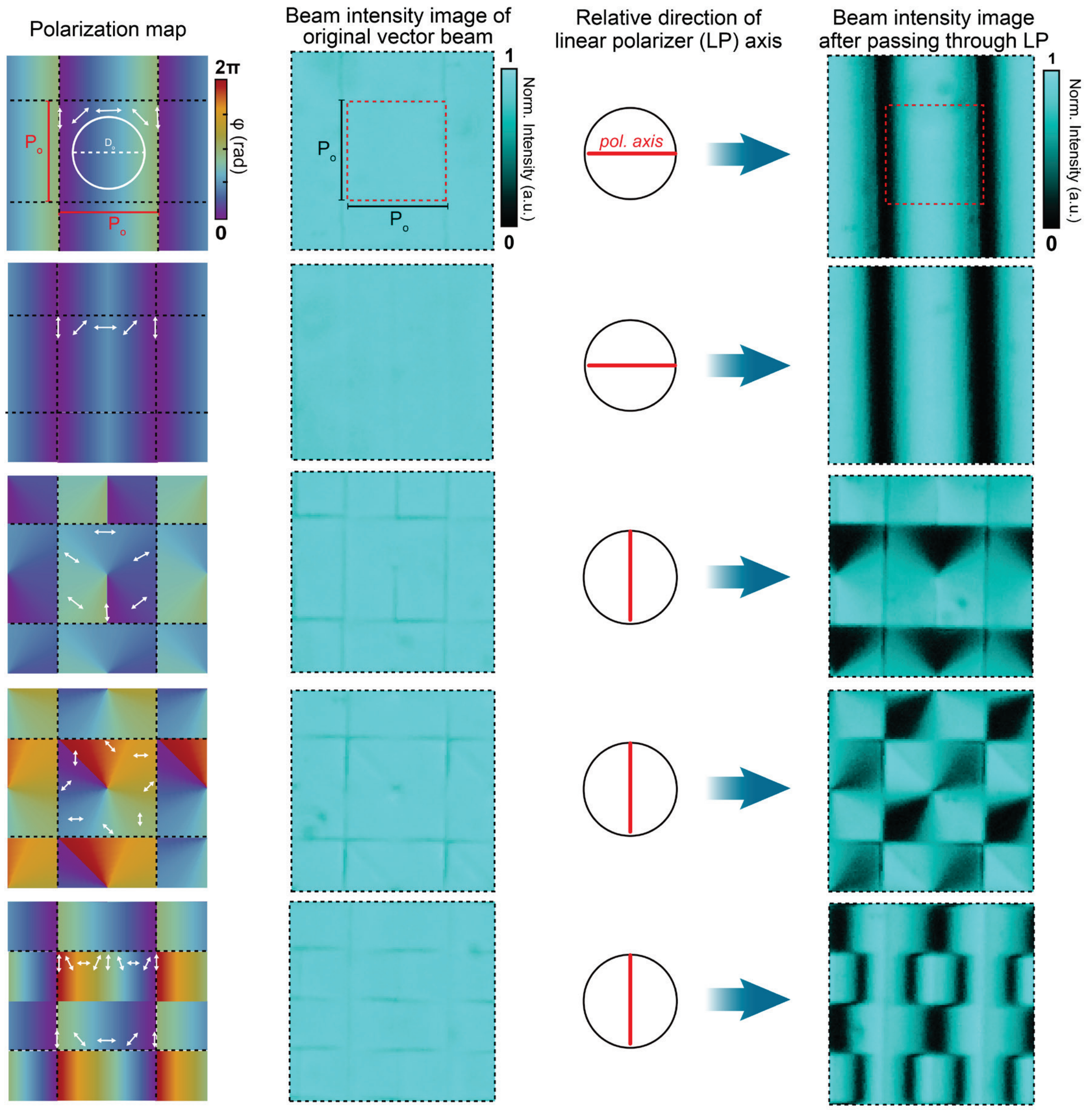

Figure S11. The qualitative characterization of the engineered beam with linear polarization filter. In the left column, the white circular line in the polarization map represents the perimeter of a single micropillar with a diameter of $D_o \approx 6.7$ µm. The white arrows provide visual indicators of the local linear polarization, as programmed by the grayscale value addressed to the SLM. To target a single micropillar in a square array, each polarization map is designed to have an area of $P_o \times P_o \approx 12 \times 12$ µm^2. The characterization was performed with setup shown in Fig. S9 a. A mirror was placed in the sample plane, and subsequently, the CCD camera was utilized to capture an intensity image that was reflected back from the mirror. The original beam intensity images were taken without a linear polarizer (LP) in front of the CCD camera , as shown in the second column. With an LP as oriented in the third column, the resulting CCD images are shown in the fourth column. In order to improve the clarity of the intensity image description, an LED 477 nm (with full width of half maximum = 29 nm) was used as the light source for this experiment.

References

1. Yadav, B.; Domurath, J.; Kim, K.; Lee, S.; Saphiannikova, M., Orientation Approach to Directional Photodeformations in Glassy Side-Chain Azopolymers. *The Journal of Physical Chemistry B* **2019,** *123* (15), 3337-3347.
2. Toshchevikov, V.; Ilnytskyi, J.; Saphiannikova, M., Photoisomerization Kinetics and Mechanical Stress in Azobenzene-Containing Materials. *Journal of Physical Chemistry Letters* **2017,** *8* (5), 1094-1098.
3. Loebner, S.; Lomadze, N.; Kopyshev, A.; Koch, M.; Guskova, O.; Saphiannikova, M.; Santer, S., Light-Induced Deformation of Azobenzene-Containing Colloidal Spheres: Calculation and Measurement of Opto-Mechanical Stresses. *Journal of Physical Chemistry B* **2018,** *122* (6), 2001-2009.
4. Loebner, S.; Yadav, B.; Lomadze, N.; Tverdokhleb, N.; Donner, H.; Saphiannikova, M.; Santer, S., Local Direction of Optomechanical Stress in Azobenzene Containing Polymers During Surface Relief Grating Formation. *Macromolecular Materials and Engineering* **2022,** *31* (n/a), 2100990.
5. Yadav, B. Modeling optical inscription of complex surface patterns in azobenzene-containing materials. PhD thesis, TU Dresden, Dresden, 2023.
6. Férec, J. Nonlinear rheology of non-spherical particle suspensions. Habilitation thesis, Universite de Bretagne-SUD, Lorient, 2016.

Table S1. The morphological measurements of different polarization map designs of engineered vector beam.

**Experiment**	**Initial Diameter D_o (µm)**	**Major axis of elongation L (µm)**	**Minor axis of elongation l (µm)**	**Related figures in main text**
Illumination using engineered vector beam with homogeneous azimuth angle of $\varphi = \pi/4$	4.0 ± 0.1	7.5 ± 0.5	2.5 ± 0.1	Fig. 3e
Illumination using engineered vector beam with checkerboard polarization pattern with periodic distribution of $\varphi_1 = \pi/4$ and $\varphi_2 = 3\pi/4$	4.0 ± 0.1	6.3 ± 0.5	2.7 ± 0.1	Fig. 3f
Gradual rotation of local linear polarization along a row of azopolymer micropillars (1st row)	4.0 ± 0.1	7.6 ± 1.1	2.4 ± 0.2	Fig. S7 (1st row)
Gradual rotation of local linear polarization along a row of azopolymer micropillars (2nd row)	4.0 ± 0.1	7.4 ± 1.1	2.6 ± 0.3	Fig. S7 (2nd row)

Movie S1. VPA dynamic model of morphological evolution of an azopolymer micropillar in the pre-patterned array (top, side, and tilted view). The model incorporates illumination parameters: homogenous linearly polarized light (blue arrow in the movie) at a wavelength of 488 nm applied over a modeled exposure time of 20 minutes. The dynamics were accelerated to reduce computational demand. The colormap represents vertical displacement: red corresponds to the initial state, while dark blue indicates the maximum downward displacement.

Movie S2. VPA dynamic model of morphological evolution of azopolymer micropillars under vectorial illumination. Top and tilted views of the modeled photo-deformation dynamics are shown for two polarization maps resulting in an inverted U-shape, and a chiral S-shape. The model incorporates experimental illumination parameters, including vector beam polarization patterns at a wavelength of 488 nm applied over a modeled exposure time of 60 seconds. The colormap represents vertical displacement: red corresponds to the initial state, while dark blue indicates the maximum downward displacement.

Movie S3. VPA dynamic model of morphological evolution of azopolymer micropillars for vectorial field-guided deformation with multi-axial symmetry. Top and tilted views of the modeled photo-deformation dynamics are shown for three polarization maps resulting in a tripetal-, quadrupetal-, and trident-shape. The model incorporates experimental illumination parameters, including vector beam polarization patterns at a wavelength of 488 nm applied over a modeled exposure time of 45 seconds for the tripetal- and quadrupetal-shape, and 90 seconds for the trident-shape. The colormap represents vertical displacement: red corresponds to the initial state, while dark blue indicates the maximum downward displacement.
